# The role of emergency surgery in hydatid liver disease

**DOI:** 10.1186/1749-7922-9-12

**Published:** 2014-01-30

**Authors:** Ali I Yahya, Hussen E Shwereif, Mustafa A Ekheil, Ahmed S Thoboot, Kalid A Algader, Fatma O Gyaed, Abdsalem S Aldarat

**Affiliations:** 1 General surgery department, Zliten Teaching Hospital and Edwaw District Hospital, Zliten, Libya

**Keywords:** Liver, Hydatid disease, Emergency surgery

## Abstract

Hepatic hydatid disease is very common in Libya. In Zliten hospital, we operated 400 patients with hepatic hydatid cysts over period of 20 years. All patients were symptomatic. Their ages varied from 3 to 85 years including 215 female and 185 male patients. Their symptoms varied from abdominal pain to abdominal mass 67 patients were admitted through Accident and Emergency Department with acute presentations including fever, skin rash, jaundice and shock with acute abdominal pain. Those 67 patients had necessary investigations, resuscitation and underwent emergency surgery. The hepatic cysts in all patients were excised, and the obstructive jaundice was cleared in those patients with obstructive jaundice. Unfortunately, one of the patients died two days after the surgery because of multiple organ failure (MOF) Morbidity was wound Infection, bile leak and recurrence rate were all reported in our series.

## Introduction

Hydatid is a disease caused by tape worm called ecchinococcous granulosis and ecchinococcous multilocularis. Hydatid disease is endemic in some regions in the world and Libya is one of the endemic countries of hydatid disease among the Middle East countries. This disease is common in sheepherder regions. Thus people who live thereare accidentally infected. Dogs are the definitive host and sheep is the intermediate host. Human gets the infection by eating contaminated vegetables by dogs' waste or by direct contact with dogs. Infection rate is higher in places where dogs are fed with infected carcasses and offal. Liver is the commonest organ involved in human as well as lung, kidneys,spleen, bones and soft tissues. Symptoms depend on the size of the cyst, the organ involved and the complications from the cyst.

The diagnosis of hydatid liver disease depends on history and clinical examination. Using imaging investigations is very valuable to confirm the diagnosis including ultrasound scan and CT scan. While serological investigations are not needed in endemic area, surgical intervention is the main way to treat hepatic hydatid cysts.

## Methods and materials

The files of the patients were reviewed retrospectively. Four hundred patients were operated at the general surgery department at Zliten hospital while sixty seven patients were operated as emergency because of their acute presentations, the research was approved by the research and ethical committee in the hospital.

67 patients were admitted through Accident and Emergency Department. One of them was shocked and admitted to the surgical ICU. All the patients came from endemic areas of hydatid disease. Routine investigations were done including complete blood count, blood group, blood sugar, urea and electrolytes. Liver function tests and ultrasound scan were done for all patients. CT scan was also performed for most of the patients. All the patients had intravenous fluid, antibiotics, analgesia and hydrocortisone.

All patients had laparotomy at same day. The operations were done under general anesthesia through right subcostal incision. Precautions were taken to prevent spillage of the cyst content. Intraoperative ultrasound was done routinely for all patients to find out the exact number of cysts. The duration of surgeries varied from one and half an hour to three hours. All the cysts were excised.

## Results

*Group A* - 45 patients were presented with abdominal pain and obstructive jaundice. Blood tests showed high bilirubin and alkaline phosphates while other blood tests were normal. The ultrasound showed hepatic hydatid cysts.

23 patients were with single big cyst with no daughter cysts. All cysts were near to the hilum with size ranges from 10 to 15 cm. In 9 patients, the cysts were in the left lobe and in the other 14 patients, they were in the right lobe. Bile ducts were normal in size.

In 22 patients, the ultrasound showed cysts with multiple daughter cysts. In twenty one of them, the cysts were in the right lobe and in one patient, the cyst was in the left lobe. The size of cysts ranges from 8 to 15 cm. MRCP was done for two patients and it showed clear communication between the cyst and the biliary tree. Preoperative ERCP was done for one patient who was in septicemia and renal impairment. 23 patients, with their cysts near the hilum, were excised and the fluid in the cyst was clear. There were no bile and no daughter cysts but only single endocyst. There was no bile duct communication and the cyst cavity was completely closed.

22 patients were with cysts at the right and the left lobe of the liver. Those cysts contain multiple daughter cysts. Some of them contain bile stained fluid. After removal of all daughter cysts, there were communications with bile ducts. With the use of the Fogarty catheter, daughter cysts and membranes came out. We flushed the bile ducts with huge amount of saline and the communication was closed with 2/0 PDS.

*Group B* - Two patients (one is male 18 years and the other is female 24 years old) were presented with swelling at anterior abdominal wall. Both the swellings were tender red, ultrasound findings in both patients showed that hydatid cyst liver communicating with the abdominal swellings which were cystic. Both patients had laparotomy and excision of the hydatid cysts and the abscess track. Both patients cured and discharged in good conditions.

*Group C *- Six patients were admitted with skin rashes all over the body. Two of them were males and the others were females ageing between 16 to 25 years. General conditions were stable and their blood pressure was within normal. On abdominal examination, there was guarding and tenderness all over the abdomen. The ultrasound examination showed huge hydatid cysts in the liver and all were in the right lobe with free fluid in the abdomen. 6 patients underwent urgent laparotomy and excision of the hydatid cysts. There was leak from the cysts into the abdominal cavity, but patients improved and discharged in good conditions.

*Group D* - One patient was admitted to Intensive Care Unit with shock. On examination, patient looked sick, pale and tachycardiac with low BP. Abdomen was guarding and rigid. The patient had resuscitation including IV fluid, IV hydrocortisone and IV analgesia. He had routine investigation and the ultrasound scan showed hydatid cyst liver in the left lobe with a lot of fluid in the abdomen. The patient had laparotomy and there was a big hydatid cyst at the left lobe which was ruptured. With excision of the cyst and cleaning of the abdominal cavity, the patient improved and discharged in good conditions.

*Group E* - Ten patients were admitted with abdominal pain and fever. They had ultrasound scan which showed hydatid cysts of the liver. In seven patients, the cysts were at the right lobe and in three patients at the left lobe. Laparotomy was performed for all of them. The cysts were full of pus, and the cyst cavity was completely cleared. Drain was inserted and the patients were covered with antibiotics. Patients improved and discharged in good conditions.

*Group F* - A female Patient was 35 years old was admitted with dyspnea and chest pain. On clinical examination, the patient looked pale and dyspnic with rapid pulse and decrease air entry on the right side of the chest.

X-ray of the chest showed that the right side was pleural effusion. The ultrasound of the abdomen showed huge hydatid cyst in the right lobe of the liver with a lot of daughter cysts in the right chest cavity. The CT scan of abdomen and chest confirmed the finding of hydatid cyst liver ruptured into chest. The patient had routine investigations and urgent surgery. The finding was a huge cyst in the right lobe of the liver full of daughter cysts and bile, ruptured into the chest cavity. Wound extended into the right thoracotomy and the hepatic cyst was excised and the fistula closed washing the pleural cavity. Drains were inserted and common bile duct was cleared from daughter cysts. The patient discharged in good conditions.

*Group G* - Two patients had laparotomy for hepatic hydatid cyst (one of them was male 18 years and the other was female 25 years old). The male Patient was diagnosed with huge right hepatic lobe hydatid cyst. MRCP showed big communication with cyst and the patient was not jaundiced. He had a laparotomy and excision of the cyst. The communication with bile duct was closed and the patient discharged in good conditions. After 10 days from the surgery, the patient came back to the hospital with abdominal pain. He looked dehydrated, sick and hypotensive. Routine investigations showed leucocytosis and raised bilirubin 3.3 mg with a slight increase of alkaline phosphates. The ultrasound showed that the abdomen was full of fluid. The patient had IV fluid, IV antibiotics and laparotomy was done. The abdomen was full of bile and it was cleaned completely. There was a leak from the cyst cavity and a big drain was inserted into the cyst cavity. Then the abdomen was closed and the patient improved and discharged in good conditions.

The female patient had laparotomy for the hydatid cyst liver. After three days, she developed severe abdominal pain. On examination, the abdomen was guarding and tender. The ultrasound showed plenty of fluid in the abdomen. The patient was sick and urgent laparotomy was done to her. The abdomen was full of bile, it was washed with plenty of saline and a big drain was inserted in the cyst cavity. And the patient improved and discharged in good conditions.

**Figure 1:**
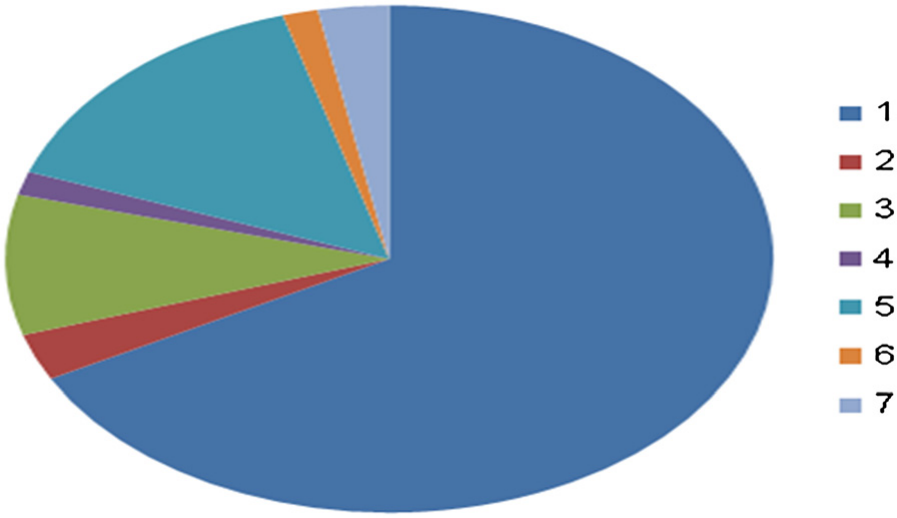
**Distribution of patients presentations.** A-45 patients with obstruction jaundice. B-2 patients with abdominal wall abscess. C-6 patients admitted with skin rash. D-One patient with anaphylactic shock. E-10 patients with liver abscess. F-1 patient with biliopleural fistula. G-2 patients with biliary peritonitis following hepatic hydatid cystectomy. Group A=1, Group B=2, Group C=3, Group D=4, Group E=5, Group F=6, Group G=7.

Sixty seven patients with hepatic hydatid cysts were admitted to our surgery ward with acute presentations included obstructive jaundice which was 11.25% of the total hydatid liver cysts operated in our institute. Six patients were presented with skin rash and acute abdominal pain was 1.25% of the total patients admitted and operated in Zlien hospital. Two patients were treated in the hospital with abdominal wall abscess which is fistula with hydatid liver cyst which was 0.5%. While ten patients were presented with liver abscess which was 2% of the total patients treated. One patient was presented with anaphylactic shock and another patient with bilipleaural fistula. Whereas another two patients were presented with peritonitis followed hepatic hydatid cyst excision. Those sixty seven patients underwent resuscitation and routine necessary investigations. All of them had urgent surgery and hepatic cysts were excised. One of the patients died after two days from the surgery due to multiple organ failure. The main hospital stay is 6 days. The morbidity noticed among 400 operated patients. 6% of them suffered from bile leak, 4% with wound infection and 2% with recurrence (Figure 1).

## Discussion and conclusion

Sixty seven patients with hepatic hydatid cysts were admitted through Accident and Emergency Department of Zliten hospital with acute symptoms. They were operated urgently and their presentations were as follow:

A. 45 patients with obstruction jaundice.

B. 2 patients with abdominal wall abscess.

C. 6 patients with skin rash.

D. 1 patient with anaphylactic shock.

E. 10 patients with liver abscess.

F. 1 patient with biliopleural fistula.

G. 2 patients with biliary peritonitis following hepatic hydatid cystectomy.

Liver is the commonest organ gets infected by ecchinococous granulosis. The cyst can be asymptomatic discovered on routine ultrasound examination of the abdomen, or symptomatic like abdominal mass or abdominal pain [1-6]. Hepatic cyst can be seen or discovered as organized cyst during investigations or other abdominal operations. In endemic areas, any patient, comes with the right upper abdominal mass, is considered infected with hydatid cyst liver until it is proved otherwise. Surgery is the main treatment for hepatic hydatid cyst. Most of our patients were treated electively. Sixty seven patients (67) with acute symptoms were operated urgently to avoid complication such as rupture which was reported in the literatures of the patients [7-14]. Different nonsurgical treatment was performed like pair technique in different centers, but none of our patients was candidate for pair technique [15-22]. Medical treatment like albendazole (antihelmentic) is used in treatment of hepatic hydatid cysts. Some centers used the medical treatment and good results were observed. But in our center, we did not report significant benefits of medical treatment. We used albendazole preoperative and postoperative to prevent recurrence and antihelmintic. Medical treatment is not beneficial for patients with acute presentation where time makes difference in management. We also used medical treatment postoperatively for our patients to prevent recurrence. Sixty seven patients who came to the Accident and Emergency Department had routine investigations and ultrasound scan, and they were treated by urgent laparotomy through the right Kocher incision. Patients of group A, with obstructive jaundice, the obstruction was done by compression on bile duct from outside and obstruction from inside by daughter cysts and parasitic membrane [23-25]. None of the patients had preoperative ERCP [26]. Only four of them had ERCP postoperatively for continuous bile leak through the drain. ERCP will not cure them but it will only clear the bile ducts from intraductal obstruction by daughter cysts or parasitic membrane and will relief the jaundice before surgery. The patient will still need surgery to cure him or her from the disease. We explored the common bile duct through the communication with the cyst for those in whom the obstructive jaundice is intraductal. But those in whom the obstruction is extra ductal the obstruction is cleared with excision of the cyst. We reviewed the literatures of the patients. Most of the patients underwent ERCP before surgery. Patients of group B suffered from abdominal wall swelling due to fistulization of superficial laying hepatic hydatid cyst. Rare presentation was also reported in literatures of patients [27,28] with biliocutaneous fistula. Patients of group C were presented with skin rash and abdominal pain due to leakage of parasitic fluid into the abdominal cavity which induced anaphylactic reaction due to antigen antibody reaction. Those patients had minimal leak where they did not go into anaphylactic shock. All of them had intravenous fluid and hydrocortisone and underwent urgent laparotomy and excision of hydatid cyst. While patients of group D were presented with anaphylactic shock due to hydatid cyst. But this was very rare. Among four hundred patients operated in our institution, only three patients had anaphylactic shock, two of them had the shock during surgery and the other patient was case reported as emergency presentation. The three patients were saved with use of intravenous adrenaline and intravenous fluid. Patients of group E were presented with liver abscess and secondary infection by bacteria. Liver abscess was expected because of high temperature [29,30] patients were sick and dehydrated. The ultrasound showed that the cyst contents were thick. Liver abscess was confirmed by surgery. All the patients had antibiotics and only three of them had blood transfusion because of low hemoglobin. The patient of group F had very rare presentation. The hepatic hydatid cyst infiltrated the diaphragm and opened in the pleura. Patients of group G had urgent surgeries. The two patients had biliary peritonitis after hepatic hydatid cyst surgery. They were diagnosed clinically and underwent urgent surgery. The usual time of surgery varies from one hour to 3 hours and the patients had excision of the cyst and closure of the cyst cavity. But none of them had liver resection although they had big drain inserted around the cyst area which left for 3 to 5 days. Emergency surgery gives quick answer to patient with acute complain. Only one patient died. She had obstructive jaundice and died because of multiple organ failure. We reported morbidity includes bile leak, wound infection, recurrence. Hospital stay was longer for patients who had emergency surgery in comparison to elective surgery.

## Conclusion

Hepatic hydatid disease can be asymptomatic or symptomatic with usual symptoms either abdominal pain or abdominal mass. We reviewed our patients admitted and treated in Zliten institution. Most of them were admitted and treated electively 67 patients were admitted through the Accident and Emergency Department. Those patients were sick and they had the investigations and treatment done urgently. They were presented with different acute presentations such as obstructive jaundice, skin rashes, anaphylactic shock, abdominal wall abscess, liver abscess, peritonitis and biliopleural fistula. All of them had urgent surgeries and could not be treated by medical or PAIR treatment. As usual, the complications from emergency surgery were expected to be more. But in this research, the rate of complications were not much different from the patients operated electively. This is due to emergency surgery done at the right time for the right patient.

## Competing interests

The authors declare that they have no competing interests.

## Authors’ contribution

All authors read and approved the final manuscript.
